# A New Disturbance Feedforward Control Method for Electro-Optical Tracking System Line-Of-Sight Stabilization on Moving Platform

**DOI:** 10.3390/s18124350

**Published:** 2018-12-10

**Authors:** Yunxia Xia, Qiliang Bao, Zidong Liu

**Affiliations:** 1 Institute of Optics and Electronics, Chinese Academy of Science, Chengdu 610209, China; control_ioe@126.com (Q.B.); yhfsir@163.com (Z.L.); 2 Key Laboratory of Optical Engineering, Chinese Academy of Sciences, Chengdu 610209, China

**Keywords:** line-of-sight stabilization, fast steering mirror, electro-optical tracking system, disturbance suppression, target tracking

## Abstract

A feedforward control was proposed based on the decoupling of target movement and disturbance from gyro signals to improve the stabilization precision of line-of-sight (LOS) for an electro-optical tracking system (EOTS) on a moving platform. Signals measured by gyros mounted on gimbal consist of target movement and disturbance. To remove target movement and obtain middle and high frequency disturbance velocity, the gyro signals were filtered by a high pass filter. The disturbance velocity was integrated into the position signal and fed forward to the inner position loop of the fast steering mirror. A detailed analysis was provided to show the proposed approach, to improve disturbance suppression performance with only slight weakening of target tracking ability. The proposed feedforward control was effectively verified through a series of comparative simulations and experiments. Besides, the method was applied in a real ship-based project.

## 1. Introduction

Electro-optical tracking systems (EOTSs) are widely used in laser communications, optical imaging, target tracking and so on. Besides, they are beginning to be increasingly mounted on vehicles, shipboard, airplanes, spacecraft and other moving platforms [1,2,3,4]. Maintaining line-of-sight (LOS) orientation toward a target will be particularly challenging when the EOTS is carried on the moving platform or when the target is highly dynamic. The stabilization of LOS is fundamental for high precision target tracking. An electro-optical tracking system consists of the two axes (or three axes) gimbal and a fast steering mirror (FSM). Gimbal serves as the main axis to realize coarse tracking and disturbance suppression in a large range, and FSM serves as the auxiliary axis to realize target fine tracking [1,5].

In the past years, a large number of works have been done to improve the disturbance rejection performance of electro-optical systems. Since both the movements of gimbal and FSM have effects on the orientation of LOS, some researchers focused on gimbal control and others focused on FSM control. In general, gimbal control usually has multi-loops, including a tracking loop, inertial rate loop and acceleration loop [6,7,8,9]. To improve the disturbance performance of gimbal, inertial rate or acceleration feedforward control based on disturbance observation or strapdown sensors were added to gimbal control. However, the control bandwidth of gimbal is severely limited to low resonance frequency, big inertia, max torque of motor. Also, this research done on gimbal only improve disturbance rejection performance at low frequencies.

Since a fast steering mirror is small in size, weight and can achieve high bandwidth, many works have been done on it to improve disturbance rejection at middle and high frequencies [10,11,12,13,14]. 

However, the signals measured by inertial sensors mounted on gimbal or FSM (FSM is also usually mounted on gimbal in an EOTS) consist of target movement achieved by gimbal coarse tracking and residue disturbance rejected by gimbal control when the system tracks dynamic target. So coarse tracking ability will be counteracted while disturbance rejection performance improved if feedback or feedforward inertial signal is sent directly to FSM, and this will be analyzed in detail in part 2. Thus, these methods were not suitable for EOTS to achieve high precision of target tracking and disturbance rejection.

In this study, to improve the disturbance rejection performance of EOTS and not weaken target tracking ability, a disturbance feedforward control was proposed based on classic control of EOTS. The key point of the proposed method was filtering the gyro signals by a high pass filter to remove target movement and obtain disturbance only. Then integrate the disturbance into position signal and feedforward it to the input of FSM inner position loop. The inner position loop of FSM provides a high bandwidth and precise control object. Experimental results prove that the proposed method has great disturbance rejection performance, primarily from 2 Hz to 100 Hz. Furthermore, the proposed method does not need mount extra sensors on FSM, so it has practical value in engineering. The rest of this paper is organized as follows. Section 2 presents theory analysis and controller design, mainly describing the control structure for EOTS with the proposed feedforward control. Section 3 shows the simulation results. Section 4 sets up experiments on an EOTS to verify this method and presents the application results. Concluding remarks are presented in Section 5.

## 2. EOTS Control Structure with Feedforward Control (CS-FC)

**Figure 1:**
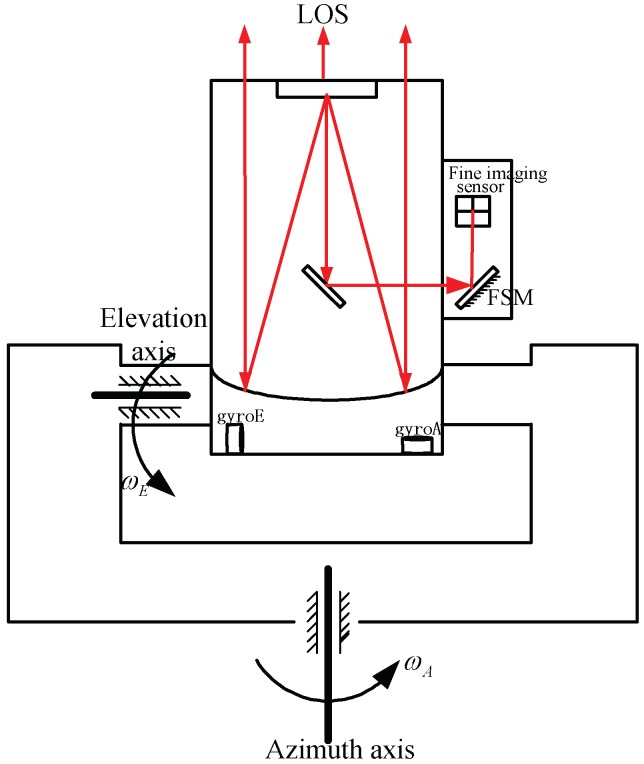
The schematic of electro-optical tracking system (EOTS) $\omega_{A}$ is the velocity of azimuth axis; $\omega_{E}$ is the velocity of elevation axis.

The sketch map of an electro-optical tracking system is shown in Figure 1. It includes a two-axis gimbal and a FSM which is mounted on the elevation axis. Gimbal was used to realize coarse target tracking and disturbance rejection; the FSM was used to realize target fine tracking [14]. Two gyros were mounted on the elevation axis and they were used to measure elevation and azimuth angler speed respectively.

**Figure 2:**
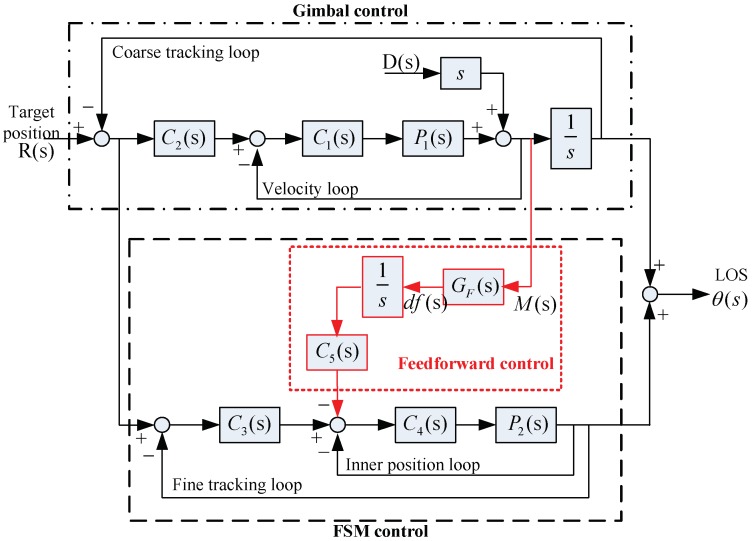
Control structure with feedforward control (CS-FC) for EOTS. $P_{1}(s)$ is the equivalent velocity control object of gimbal, it may include acceleration loop and current loop; $P_{2}(s)$ is the position control object of fast steering mirror(FSM); $C_{1}(s)$ is the gimbal velocity loop controller; $C_{2}(s)$ is the gimbal tracking loop controller; $C_{3}(s)$ is the FSM tracking loop controller; $C_{4}(s)$ is the FSM inner position loop controller; $C_{5}(s)$ is the feedforward controller; $G_{F}(s)$ is the filter; D(s) is the disturbance angle; R(s) is the target position;θ(s) is the line-of-sight (LOS) position.

Classic control structure (CCS) for EOTS and the control structure with feedforward control (CS-FC) is shown in Figure 2. The red part is the proposed feedforward control, and except the red part it is the classic control structure. In the classic control structure, all the gimbal velocity loops, coarse tracking loops and fine tracking loops can reject disturbance. However, the bandwidth of gimbal velocity loop primarily limited to low mechanical resonance frequency and both the coarse tracking and fine tracking loop bandwidth are severely limited due to large time delay of the imaging sensors. Accordingly, disturbance feedforward control was designed based on CCS to expand disturbance rejection bandwidth.

Define as:

$G_{1}(s)=\frac{C_{1}(s)P_{1}(s)}{1+C_{1}(s)P_{1}(s)}$ is the close loop transfer function of gimbal velocity loop.
$G_{2}(s)=\frac{C_{4}(s)P_{2}(s)}{1+C_{4}(s)P_{2}(s)}$ is the close loop transfer function of FSM inner position loop.
$W_{vel}(s)=C_{1}(s)P_{1}(s)$ is the open loop transfer function of gimbal velocity loop.
$W_{CTrack}(s)=C_{2}(s)G_{1}(s)\frac{1}{s}$ is the open loop transfer function of coarse tracking loop.
$W_{FTrack}(s)=C_{3}(s)G_{2}(s)$ is the open loop transfer function of fine tracking loop.

According to Figure 2, it is easy to obtain the transfer function of CCS.

(1) $$\begin{array}{cc} \theta_{CCS}(s) & =\left(\frac{W_{CTrack}(s)}{1+W_{CTrack}(s)}+\frac{1}{1+W_{CTrack}(s)}\times \frac{W_{FTrack}(s)}{1+W_{FTrack}(s)}\right)R(s)\\ & +\left(\frac{1}{1+W_{CTrack}(s)}\times \frac{1}{1+W_{FTrack}(s)}\times \frac{1}{1+W_{vel}(s)}\right)D(s) \end{array}$$

(2) $$\begin{array}{cc} \theta_{CS-FC}(s) & =\left(\frac{W_{CTrack}(s)}{1+W_{CTrack}(s)}+\frac{1}{1+W_{CTrack}(s)}\times \frac{W_{FTrack}(s)}{1+W_{FTrack}(s)}-\frac{W_{CTrack}(s)}{1+W_{CTrack}(s)}\times \frac{G_{2}(s)G_{F}(s)C_{5}(s)}{1+W_{FTrack}(s)}\right)R(s)\\ & +\frac{1-G_{2}(s)G_{F}(s)C_{5}(s)}{\left(1+W_{CTrack}(s)\right)\left(1+W_{FTrack}(s)\right)\left(1+W_{vel}(s)\right)}D(s) \end{array}$$

The LOS output transfer function of CS-FS is given by Equation (2).

### 2.1. Feedforward Control Design

According to Figure 2 M(s) is the signal which is measured by gyro.

(3) $$M(s)=\frac{1}{1+W_{CTrack}(s)}\times \frac{1}{1+W_{vel}(s)}sD(s)+\frac{W_{CTrack}(s)}{1+W_{CTrack}(s)}R(s)$$

Equation (3) shows that M(s) contains two parts, one is the residual disturbance rejected by gimbal control and the other is the target movement achieved by coarse tracking. So, $G_{F}(s)$ was used to filter M(s) to obtain disturbance only. Disturbance usually has a wide bandwidth, from low frequency up to hundreds Hz. Comparing with wide bandwidth of disturbance, coarse tracking control bandwidth is very low. Thus, the $G_{F}(s)$ was designed as a high pass filter, and by appropriate designing the bandwidth of $G_{F}(s)$, $G_{F}(s)\frac{W_{FTrack}(s)}{1+W_{FTrack}(s)}R(s)\approx 0$.

From Equation (2), it is easy to see that if $C_{5}(s)={\left(G_{2}(s)G_{F}(s)\right)}^{-1}$, CS-FC can complete compensate disturbance. However, in engineering applications it is hard to realize complete compensation since the inverse of $G_{2}(s)$ usually contains differentiator. Also, $G_{F}(s)\approx 1$ at middle and high frequency, so the feedforward controller was designed as:

(4) $$C_{5}(s)={\left(G_{2}(s)\right)}^{-1}\times \frac{1}{{\left(Ts+1\right)}^{n}}$$

It is the inverse of $G_{2}(s)$ that multiplies a low pass filter, where n denotes the order difference between numerator and denominator and the bandwidth of low pass filter extends three hundred Hz. By the design of $C_{5}(s)$ as Equation (4) shows, $C_{5}(s)$ is nearly equal to the inverse of $G_{2}(s)$ at a very wide bandwidth.

### 2.2. Target Tracking Performance Analysis

The error between LOS and target is θ(s)−R(s), consisting of target tracking error and disturbance rejection error. From Equation (1) it is easy to get the target tracking error of CCS, it is shown in Equation (5).

(5) $${Error}_{CCS}^{R}(s)=\frac{1}{1+W_{CTrack}(s)}\times \frac{1}{1+W_{FTrack}(s)}R(s)$$

According Equation (5), it can be known that the target tracking ability of CCS is the product of coarse tracking loop and fine tracking loop.

As previously analyzed $G_{F}(s)\frac{W_{FTrack}(s)}{1+W_{FTrack}(s)}R(s)\approx 0$. Substituting this into Equation (2), it yields the target tracking transfer function of CS-FC in Equation (6)

(6) $$\theta_{CS-FC}^{R}(s)\approx \left(\frac{W_{CTrack}(s)}{1+W_{CTrack}(s)}+\frac{1}{1+W_{CTrack}(s)}\times \frac{W_{FTrack}(s)}{1+W_{FTrack}(s)}\right)R(s)$$

According Equation (6), the target tracking error of CS-FC is presented as:

(7) $$Error_{CS-FC}^{R}(s)=R(s)-\theta_{CS-FC}^{R}(s)\approx \frac{1}{1+W_{CTrack}(s)}\times \frac{1}{1+W_{FTrack}(s)}R(s)$$

Compared Equations (5) and (7), the target tracking performance of CS-FC is almost the same as that of CCS.

If feedforward the gyro signals directly to FSM, it means $G_{F}(s)=1$ in Figure 2. Then according Equation (2), the target tracking transfer function and target tracking error transfer function are presented in Equations (8) and (9):

(8) $$\begin{array}{cc} {\overset{\wedge }{\theta }}_{CS-FC}^{R}(s) & \approx \left(\frac{W_{CTrack}(s)}{1+W_{CTrack}(s)}+\frac{1}{1+W_{CTrack}(s)}\times \frac{W_{FTrack}(s)}{1+W_{FTrack}(s)}-\frac{W_{CTrack}(s)}{1+W_{CTrack}(s)}\times \frac{1}{1+W_{FTrack}(s)}\right)R(s)\\ & =\frac{W_{FTrack}(s)}{1+W_{FTrack}(s)}R(s) \end{array}$$

(9) $${\overset{\wedge }{Error}}_{CS-FC}^{R}(s)=R(s)-{\overset{\wedge }{\theta }}_{CS-FC}^{R}(s)\approx \frac{1}{1+W_{FTrack}(s)}R(s)$$

Obviously, according to Equations (8) and (9), the coarse tracking ability is countered if the gyro signals are directly fed forward to FSM. Therefore, target movement and disturbance must be decoupled from gyro signals.

### 2.3. Disturbance Rejection Performance Analysis

From Equation (1) it is easy to get the disturbance rejection error of CCS, it is shown in Equation (10).

(10) $${Error}_{CCS}^{D}(s)=\frac{1}{1+W_{CTrack}(s)}\times \frac{1}{1+W_{vel}(s)}\times \frac{1}{1+W_{FTrack}(s)}D(s)$$

According to Equation (2), the disturbance rejecting error of CS-FC is:

(11) $${Error}_{CS-FC}^{D}(s)=\frac{1-C_{5}(s)G_{2}(s)G_{F}(s)}{\left(1+W_{CTrack}(s)\right)\left(1+W_{FTrack}(s)\right)\left(1+W_{vel}(s)\right)}D(s)$$

At low frequency, $G_{F}(s)\approx 0$, so the disturbance rejection performance of CS-FC is:

(12) $${Error}_{CS-FC}^{D}(s)=\frac{1}{\left(1+W_{CTrack}(s)\right)\left(1+W_{FTrack}(s)\right)\left(1+W_{vel}(s)\right)}D(s)$$

At middle and high frequency, $G_{F}(s)\approx 1$, so the disturbance rejection performance of CS-FC is:

(13) $${Error}_{CS-FC}^{D}(s)=\frac{1-C_{5}(s)G_{2}(s)}{\left(1+W_{CTrack}(s)\right)\left(1+W_{FTrack}(s)\right)\left(1+W_{vel}(s)\right)}D(s)$$

Equations (10) and (13) show that disturbance rejection performance of CS-FC is the same as CCS at low frequency. But the numerator of Equation (13) becomes to $1-C_{5}(s)G_{2}(s)$ and $C_{5}(s)G_{2}(s)\approx 1$, so CS-FC improves disturbance rejection performance at middle and high frequency.

## 3. Simulation

**Figure 3:**
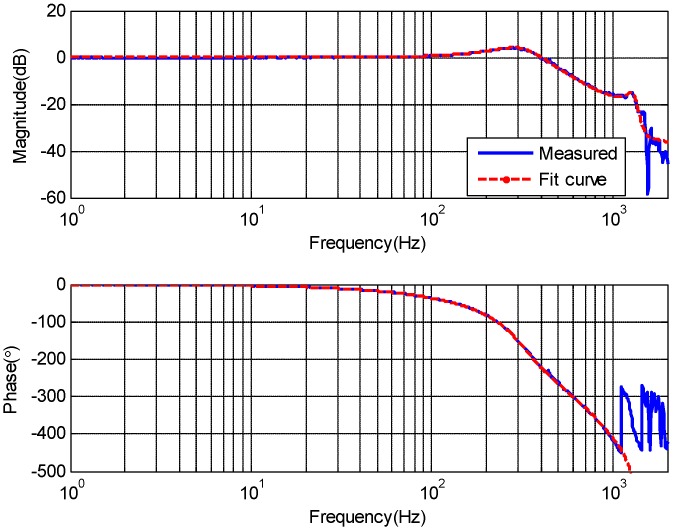
The close loop characteristic of the FSM inner position loop.

**Figure 4:**
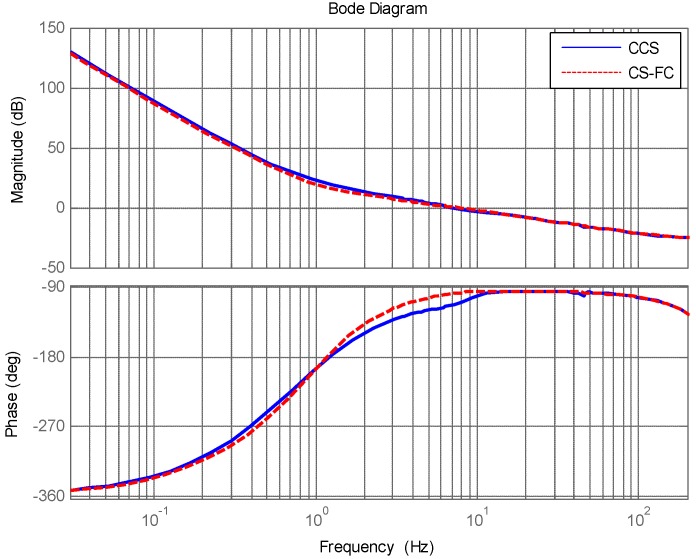
Target tracking open loop response of CCS and CS-FC.

**Figure 5:**
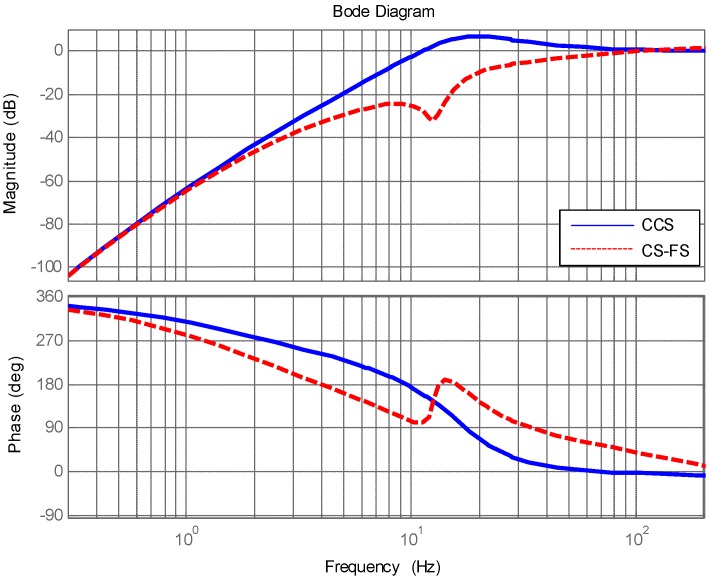
Disturbance rejection performance of classic control structure (CCS) and CS-FC.

**Figure 6:**
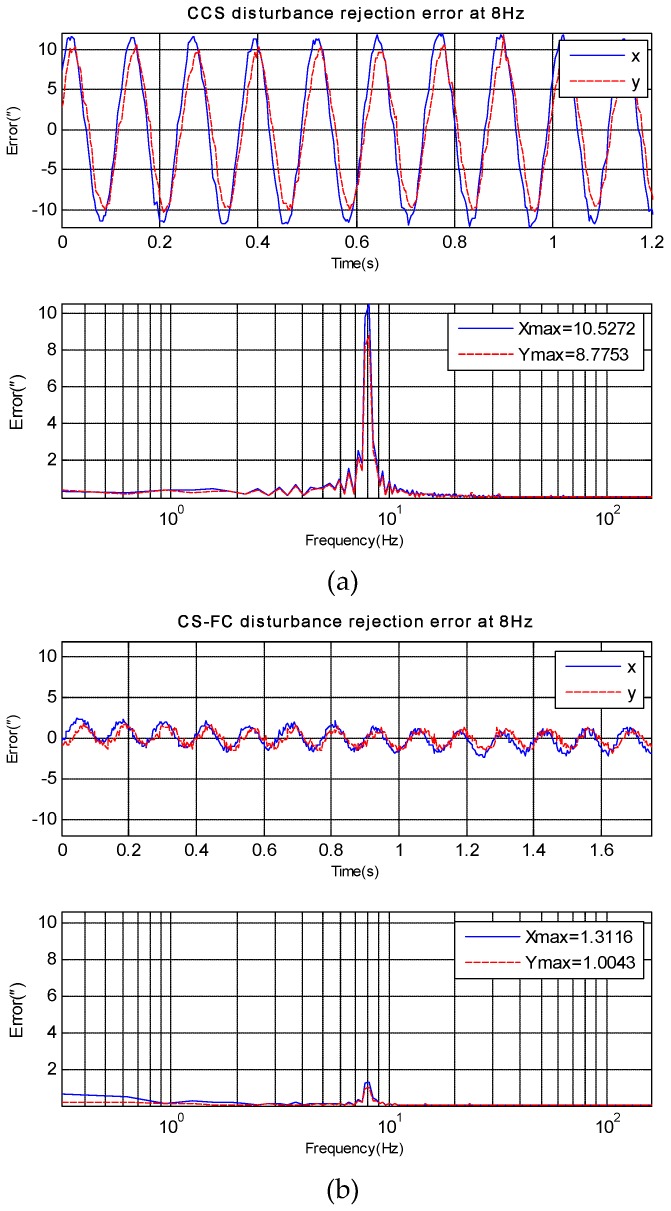
*X*-axis and *Y*-axis disturbance rejection error at 8 Hz. (**a**) disturbance rejection error of CCS; (**b**) disturbance rejection error of CS-FC.

**Figure 7:**
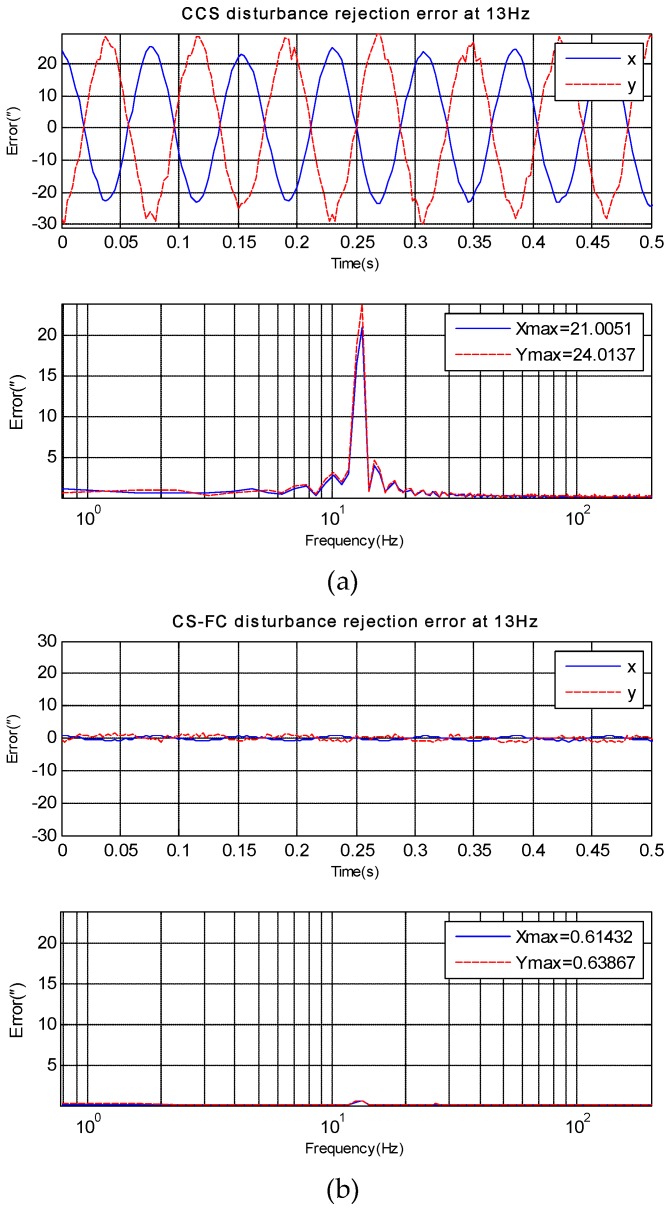
*X*-axis and *Y*-axis disturbance rejection error at 13 Hz. (**a**) disturbance rejection error of CCS; (**b**) disturbance rejection error of CS-FC.

**Figure 8:**
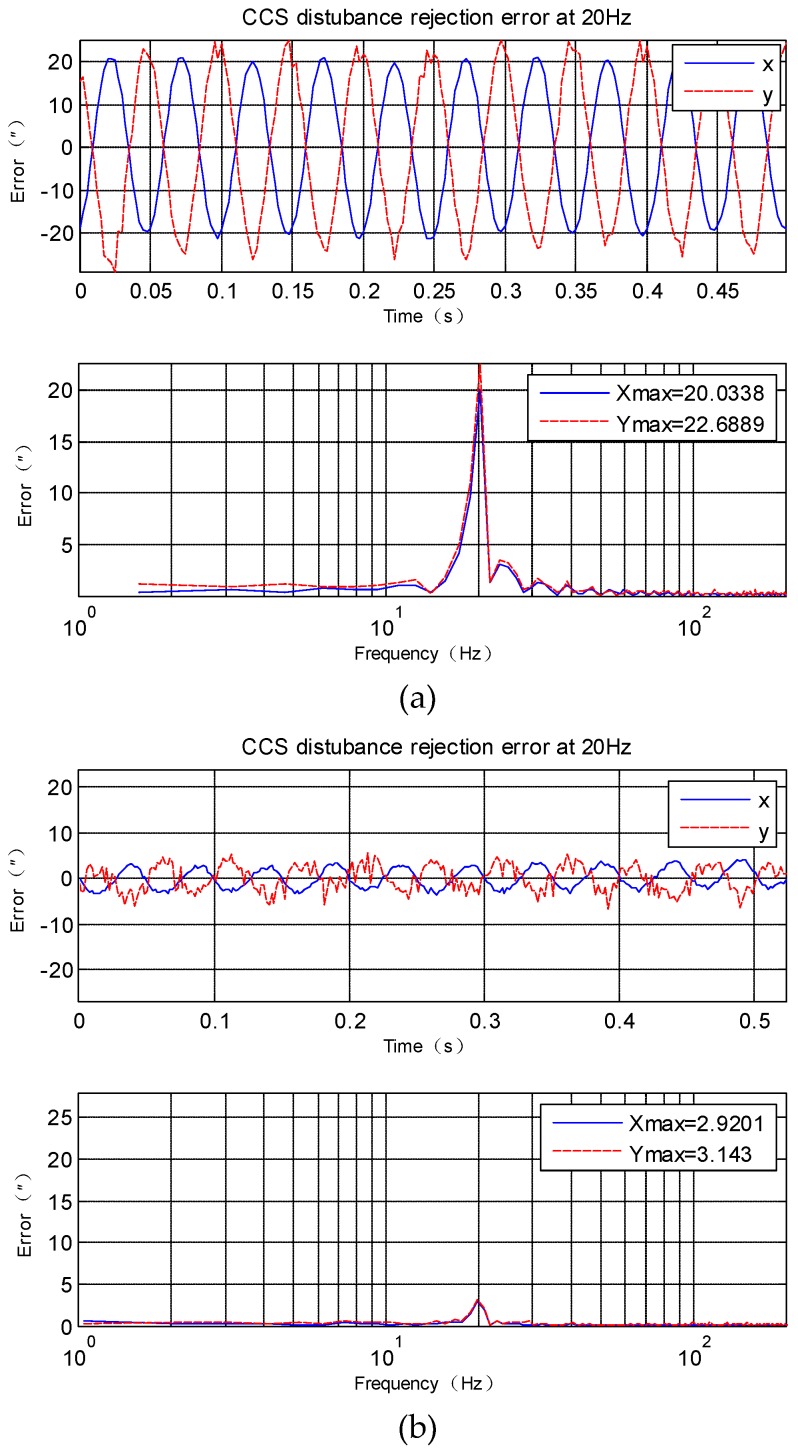
*X*-axis and *Y*-axis disturbance rejection error at 20 Hz. (**a**) disturbance rejection error of CCS; (**b**) disturbance rejection error of CS-FC.

**Figure 9:**
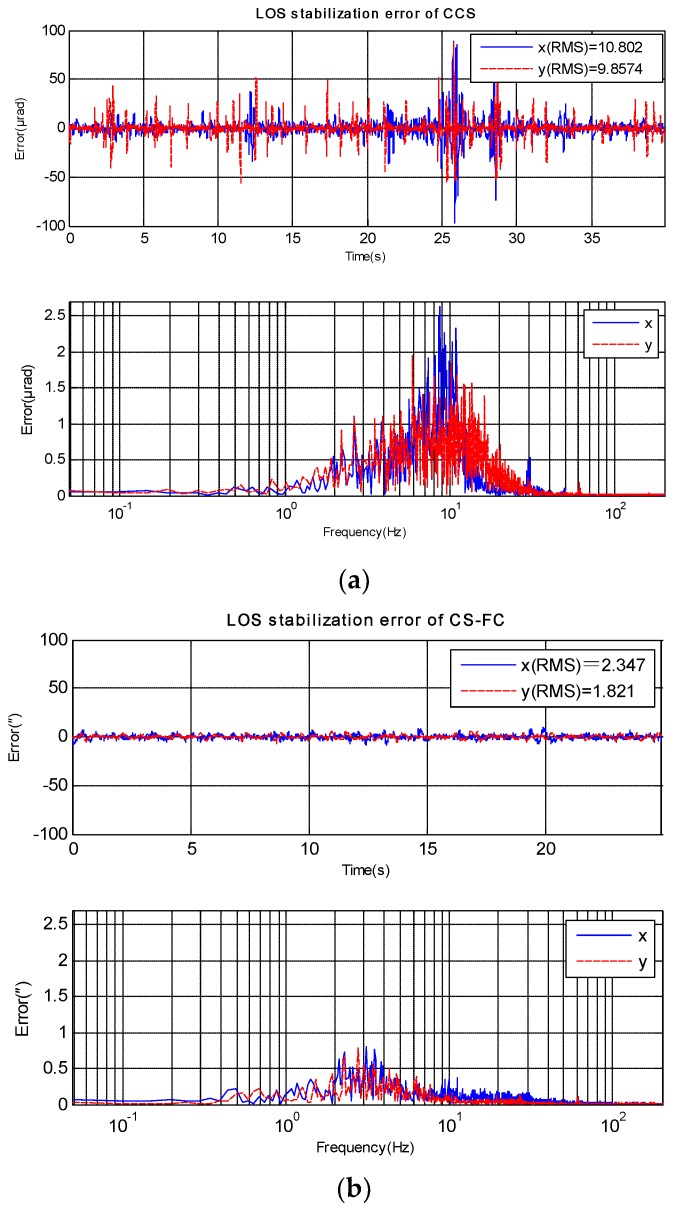
Shipboard ETOS LOS stabilization error(*X*-axis and *Y*-axis). (**a**) stabilization error of CCS; (**b**) stabilization error of CS-FC.

Simulations have been done based on the model of a real EOTS. Here, it primarily gives the design about disturbance feedforward control because other control part of CS-FC is the same as CCS except the disturbance feedforward control. The close loop characteristic of FSM inner position loop response was measured by spectral analyzer from 1 Hz to 2000 Hz is shown in Figure 3. The upper parts is the magnitude-frequency characteristics, the lower parts is the phase-frequency characteristics, and this same for Figure 4, Figure 5, Figure 6, Figure 7, Figure 8 and Figure 9.

The transfer function of the FSM inner position loop was obtained by fitting the measurement data. The transfer function is:

(14) $$P_{2}(s)=\frac{{\omega_{n}}^{2}}{s^{2}+2\xi_{n}\omega_{n}s+{\omega_{n}}^{2}}\times \frac{{\omega_{2}}^{2}(s^{2}+2\xi_{1}\omega_{1}s+{\omega_{1}}^{2})}{{\omega_{1}}^{2}(s^{2}+2\xi_{2}\omega_{2}s+{\omega_{2}}^{2})}\times e^{-\tau s}$$

where $\omega_{n}=320\times 2\pi$, $\xi_{n}=0.33$, $\omega_{1}=1500\times 2\pi$, $\xi_{1}=0.1$, $\omega_{2}=1300\times 2\pi$, $\xi_{2}=0.06$, τ=0.00068.

Based on the previous analysis and the FSM inner position loop transfer function, the feedforward controller was designed as:

(15) $$C_{5}(s)=\frac{s^{2}+1327s+4043000}{2.587s^{2}+6468s+4043000}$$

The filter $G_{F}(s)$ was designed as:

(16) $$G_{F}(s)=\frac{s^{2}}{\left(s^{2}+11.26s+74.26\right)}$$

Figure 4 shows the target tracking open loop response of CCS and CS-FC. Obviously, the target tracking ability of CS-FC is almost the same as the CCS. The disturbance feedforward control in CS-FC does not weaken target tracking performance by adding $G_{F}(s)$ in the control structure, and it was designed as Equation (16) shows.

The disturbance rejection performance simulation results of CCS and CS-FC are shown in Figure 5. The disturbance rejection performance of CS-FC was primarily improved from 2 Hz to 100 Hz compared with that of CSS.

## 4. Experiments and Applications Results

Experiments have been done on an EOTS, and the sketch map of the system is shown in Figure 1. The disturbance rejection of CCS and CS-FC in *X* axis and *Y* axis can be measured by fine imaging sensor when disturbance platform is moving at a different frequency. Then three experiments in different frequency disturbance are compared in Figure 6, Figure 7 and Figure 8. Since the gimbal is so heavy, and the disturbance can’t vibrate precisely at too much high frequency, here the max disturbance frequency is given, namely 20 Hz. The stabilization error of CCS and CS-FC and improvement of CS-FC are listed in Table 1. According to experimental results, the CS-FC achieved the best disturbance rejection at 13 Hz, and both the *X*-axis and *Y*-axis improved more than 30dB at this frequency compared with the performance of CCS.

Furthermore, CS-FC was employed by a ship-based EOTS, and the LOS stabilization precision in real marine environment was tested. The LOS error of CCS and CS-FC are shown in Figure 9and Table 2. The result shows that CS-FC effectively improved LOS stabilization precision. Compared to the frequency spectrum of Figure 9a,b it is clearly seen that the disturbance rejection performance of CS-FC is almost equal to CCS below 2 Hz and has been improved above2 Hz, especially from 5 Hz to 20 Hz, where the main errors of CCS occur.

## 5. Conclusions

This paper focused on the design of disturbance feedforward control to improve disturbance rejection performance and not affect target tracking ability. For this end, CS-FC was proposed. Its main idea is to decouple target movement and disturbance from gyro signals mounted on gimbal and just feedforward disturbance to the inner position loop of FSM. According to the results of the experiments, this technique effectively improves disturbance rejection performance compared with the classical control mode. Furthermore, the proposed method was applied to a ship-based project and achieved much better LOS stabilization precision than CCS.

Future study will focus on improving disturbance rejection ability from low frequencies up to several Hz. This is very challenging as it must decouple target movement and disturbance, even when they are at the same frequency.

## Figures and Tables

**Table 1:** The disturbance rejection performance of CCS and CS-FC.

DisturbanceFrequency	Rejection Error (″)				Improvement of CS-FC (dB)	
	CCS		CS-FC			
	X-Axis	Y-Axis	X-Axis	Y-Axis	X-Axis	Y-Axis
**8 Hz**	10.5	8.8	1.3	1.0	18.1	18.8
**13 Hz**	21.0	24.0	0.61	0.64	30.7	31.5
**20 Hz**	20.0	22.7	2.9	3.1	16.8	17.3

**Table 2:** Shipboard LOS stabilization error of CCS and CS-FC.

Control Method	PPV: Peak to Peak Value (μrad)		RMS: Root Mean Square (μrad)		
	X-Axis	Y-Axis	X-Axis	Y-Axis	Total
**CCS**	186.3	144.7	10.8	9.6	14.5
**CS-FC**	18.2	13.2	2.3	1.8	2.9

## References

[1] Ulich B.L. (1988). Overview of acquisition, tracking, and pointing system technologies. Proceedings of the SPIE Volume 0887, Acquisition, Tracking, and Pointing II, Los Angeles, CA, USA, 3 May 1988.

[2] Barker K.W. (1999). Airborne and Space-Based Lasers: An Analysis of Technological and Operational Compatibility.

[3] Borrello M. A Multi Stage Pointing Acquisition and Tracking (PAT) Control System Approach for Air to Air Laser Communications. Proceedings of the American Control Conference.

[4] Ang C.N. (2012). Analysis of High Energy Laser Weapon Employment from a Navy Ship. Master’s Thesis.

[5] Ma J.G. (1989). Basic technology problem in acquisition, tracking and pointing system. Opto-Electron. Eng..

[6] Miller R., Mooty G. (1988). Gimbal system configurations and line-of-sight control techniques for small UAV applications. Proceedings of SPIE Volume 8713, Airborne Intelligence, Surveillance, Reconnaissance (ISR) Systems and Applications X, Baltimore, MD, USA, 29 April–3 May 2013.

[7] Hilkert J.M. (2008). Inertially Stabilized Platform Technology: Concepts and principles. IEEE Control Syst. Mag..

[8] Wang H.G., Williams T.C. (2008). Strategic Inertial Navigation Systems: High-Accuracy Inertially Stabilized Platforms for Hostile Environments. IEEE Control Syst. Mag..

[9] Wang Q., Cai H.-X., Huang Y.-M., Ge L., Tang T., Su Y.-R., Liu X., Li J.-Y., He D., Du S.-P. (2016). Acceleration feedback control (AFC) enhanced by disturbance observation and compensation (DOC) for high precision tracking in telescope systems. Res. Astron. Astrophys..

[10] Hilkert J.M. (1988). A comparison of inertial line-of-sight stabilization techniques using mirrors. Proceedings Volume 5430, Acquisition, Tracking, and Pointing XVIII, Orlando, FL, USA, 13–15 April 2004.

[11] Tang T., Huang Y., Fu C., Liu S. (2009). Acceleration feedback of a CCD-based tracking loop for fast steering mirror. Opt. Eng..

[12] Tian J., Yang W., Peng Z., Tang T., Li Z. (2016). Application of MEMS accelerometers and gyroscopes in fast steering mirror control systems. Sensors.

[13] Ren W., Deng C., Mao Y., Ren G. (2017). Virtual velocity loop based on MEMS accelerometers for optical stabilization control system. Opt. Eng..

[14] Deng C., Mao Y., Ren G. (2016). MEMS Inertial Sensors-Based Multi-Loop Control Enhanced by Disturbance Observation and Compensation for Fast Steering Mirror System. Sensors.

